# Can Sleep Quality be Associated With Positional Vertigo in Children With Dizziness?

**DOI:** 10.1002/brb3.70654

**Published:** 2025-07-09

**Authors:** Busra Umame Sahin, Tugce Gurel Soylemez, Gorkem Ertugrul

**Affiliations:** ^1^ Institute of Health Sciences, Department of Audiology Hacettepe University Ankara Turkey; ^2^ Faculty of Health Sciences, Department of Audiology Hacettepe University Ankara Turkey

**Keywords:** balance, child, dizziness, sleep, vertigo

## Abstract

**Objective:**

Vertigo and dizziness are more common than expected in children and adolescents. The sleep quality of children with dizziness is unknown. This study aimed to investigate the relationship between sleep quality, vertigo severity, and duration of dizziness in children with positional vertigo.

**Methods:**

This study consisted of 26 children with dizziness (19 female, 7 male, and mean age 14.38 ± 2.02 years). Positional tests were performed using video Frenzel glasses. The participants' sleep quality was assessed using the Sleep Disturbance Scale for Children (SDSC), the severity of vertigo was assessed using the Visual Analog Scale (VAS), and the functional balance was measured using the Pediatric Balance Scale.

**Results:**

While 58% of children with dizziness (15/26) had good sleep quality, 42% (11/26) had poor sleep quality. A significant positive correlation was found between the dizziness durations and SDSC scores (*p* = 0.03, *r_s_*: 0.42). Positional vertigo was significantly more frequent in children with dizziness who had poor sleep quality compared to those with good sleep quality (*p* < 0.001). Regarding the SDSC subscales, significant positive correlations were observed between dizziness duration and both the “Disorders of Initiating and Maintaining Sleep” subscale (*p* = 0.040, *r_s_*: 0.40) and the “Excessive Somnolence” subscale (*p* = 0.030, *r_s_*: 0.40).

**Conclusion:**

Assessing sleep quality is an essential component of the clinical vestibular evaluation in children with dizziness. Inquiring about sleep patterns and disturbances can provide valuable insights into factors affecting their overall well‐being. Enhancing sleep quality may contribute to a reduction in the frequency and severity of vertigo and dizziness symptoms, thereby improving daily functioning and overall quality of life.

## Graphical abstract

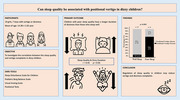

## 1 Introduction

Vertigo, as a complex and subjective symptom, is now believed to be more prevalent among children and adolescents than previously thought (Brodsky et al. 2020; Lee et al. 2017). One of the main reasons for this underestimation is that children may have difficulty articulating what they feel, or they may describe the symptom in a way that is vague or inconsistent with clinical terminology. As a result, vertigo in this age group can easily go unrecognized or be misattributed to other conditions. Delayed postural control and lack of coordination in children may result from vestibular deficits, vertigo, and dizziness. To ensure successful treatment, it is important to identify the triggers of dizziness and vertigo. These triggers are the same in children and adults and can include body or head movements, coughing, sneezing, insomnia, and psychosocial stress (Jahn 2016).

Maintaining balance and movement in humans is a sensory integration process involving the coordination of stimuli from the visual, vestibular, and proprioceptive systems (DeAngelis and Angelaki 2012). The integration of these stimuli from the structural and sensory systems occurs in the central nervous system and is influenced by various factors, including sleep (Bae et al. 2022).

The physiological necessity of sleep for humans is essential as it governs numerous vital bodily processes, including hormone equilibrium, memory, organization, and focus (Tetych et al. 2018). The hypothalamus controls the balance between sleep and wakefulness (Tetych et al. 2018). Connections have been identified between the suprachiasmatic hypothalamic nucleus structures of the hypothalamus and the vestibular nuclei and end organs, contributing to the balance between sleep and wakefulness (Besnard et al. 2018). Disruption of any of these systems or detection of incompatible stimuli between systems can cause symptoms such as vertigo, dizziness, nausea, vomiting, blurred vision, nystagmus, head and neck instability, changes in heart rate, and sleep problems (Alpini et al. 2014; Sugaya et al. 2017). The cause‐and‐effect relationship between vestibular pathologies and sleep disturbance is not fully known. Sleep problems may be a cause or consequence of vertigo and dizziness (Mutlu and Topcu 2022). The vestibular system provides information about the head's position and may influence the onset and duration of sleep (Wiener and Taube 2005).

Sleep quality in children can be evaluated using various objective and subjective methods. Among the subjective tools, the Sleep Disturbance Scale for Children (SDSC) stands out as a valid and reliable instrument specifically designed to assess sleep disturbances in pediatric populations (Ağadayı et al. 2020). The SDSC evaluates multiple dimensions of sleep, including disorders of initiating and maintaining sleep, sleep‐wake transition disorders, disorders of arousal, sleep‐related breathing disorders, disorders of excessive somnolence, and sleep hyperhidrosis. Its comprehensive structure allows for a detailed understanding of the child's sleep profile, making it a suitable measure for exploring the relationship between sleep disturbances and other clinical symptoms such as dizziness or vertigo.

In clinical practice, the impact of sleep disorders on pediatric patients who experience dizziness is often underestimated. We believe that vestibular symptoms can negatively affect sleep quality in children and that the degree of these effects may vary depending on the severity and duration of the symptoms. Therefore, this study aims to investigate the relationships between sleep quality, the severity of dizziness, and the duration of dizziness.

## 2 Materials and Methods

### 2.1 Participants

Participants who experienced vertigo or dizziness between the ages of 6 and 16 were included in this study. G*Power 3.1 software was used to calculate the sample size. Using a one‐tailed *t*‐test for the difference between two independent means, with an effect size of 1.02, a power of 80%, and a significance level of 0.05, a minimum total sample size of 26 was required for the study (Aydogan et al. 2023). Of the 26 children involved in the study, 19 (73.1%) were girls and 7 collection (26.9%) were boys. The inclusion and exclusion criteria of the study are outlined as follows.

### 2.2 Inclusion Criteria

Aged between 9 and 16 years.

Presence of complaints of vertigo or dizziness.

Normal hearing levels.

Written and verbal informed consent obtained from parents or legal guardians.

### 2.3 Exclusion Criteria

Children diagnosed with any hearing loss or those using hearing aids or cochlear implants.

Children diagnosed with sleep apnea.

Children with systemic diseases that may affect sleep quality, such as diabetes mellitus, neurological disorders like epilepsy, and chronic respiratory diseases such as asthma.

The mean age of the children was 14.38 ± 2.02 years (min–max: 9–16 years). Twelve of them were born by cesarean section, whereas 14 were born normally. According to gross motor development, the mean age for sitting without support was 5.53 ± 0.90 months, and the mean age for independent walking was 11.73 ± 1.40 months. The demographic information of the participants is presented in Table 1. All children with dizziness had normal hearing in this study.

**TABLE 1:** Demographic information of the participants.

Characteristics	*x̄* ± SD
Age	14.38 ± 2.02 (range 9–16 years)
Height (cm)	162 ± 11.09
Weight (kg)	57.80 ± 16.42
Birth weight (g)	3091.30 ± 672.43
Birth type (*n*)	
Normal	14
Cesarean	12
Gross motor development	
Independent walking (month)	11.73 ± 1.40
Sitting without support (month)	5.53 ± 0.90
*n*	26

Abbreviation: SD, standard deviation.

Verbal and written approval was obtained from each child and parent. All patients who agreed to participate in the study provided written informed consent. This study was conducted at the Audiology Unit of Hacettepe University Hospitals and was approved by the Non‐Interventional Ethics Board of Hacettepe University (GO 23/439).

### 2.4 Data Tools

In this study, detailed medical histories were collected from all patients. All children underwent assessments for spontaneous nystagmus and positional tests to evaluate their vestibular function. The Sleep Disturbance Scale for Children (SDSC) was utilized to identify sleep disturbances in children experiencing dizziness. The Visual Analog Scale (VAS) was employed to measure the severity of vertigo. Additionally, self‐reports or parental reports were used to determine the duration of dizziness. The Pediatric Balance Scale (PBS) was applied to assess the functional balance skills of children with dizziness.

### 2.5 Sleep Disturbance Scale for Children

The Turkish edition of the Sleep Disturbance Scale for Children was used to reliably and quickly evaluate children's sleep quality (Ağadayı et al. 2020). Based on parental feedback, this scale assesses sleep problems in the last 6 months. The scale consists of 26 items; each item is scored between 1 and 5 points. Higher scores indicate the presence of a sleep disorder. This scale was developed to assess various types of sleep disorders in children and to provide a general measure of sleep disruption for research and clinical screening purposes. Bruni et al. (1996) used factor analysis to classify sleep disorders into six groups representing some of the most common sleep issues in children and children. These include hyperhidrosis (nighttime sweating), arousal and nightmare disorders, sleep‐wake transition disorders, hypersomnolence disorders, and sleep initiation and maintenance disorders. In this study, a cutoff point for sleep quality score was established at 42. The numerical SDSC data were divided into two groups: SDSC score ≤ 42 indicates good sleep quality, while SDSC score > 42 indicates poor sleep quality. (Bruni et al. 1996). The scale items demonstrated acceptable internal consistency (Cronbach's *α* = 0.84) (Agca et al. 2021).

### 2.6 Visual Analog Scale

The Visual Analog Scale was first developed in 1921 to assess pain (Hayes and Patterson 1921). However, it is also commonly used to evaluate dizziness objectively (Toupet et al. 2011; Hunter et al. 2022). Patients were asked to rate the severity of vertigo on a VAS ranging from 0 (no dizziness) to 10 (very severe dizziness). The VAS vertigo severity score was converted to the categorical data: VAS score ≤ 5: low vertigo severity and VAS score > 5: high vertigo severity.

### 2.7 Pediatric Balance Scale

The participants' functional balance skills were assessed using the PBS, which consists of 14 items evaluated on a scale of 0–4 points. Higher scores indicate better balance performance. The internal consistency of the items is acceptable, with Cronbach's *α* ± 0.85 (Erden et al. 2021).

### 2.8 Positional Tests

During the positional testing, the Dix‐Hallpike, supine roll, and deep head‐hanging maneuvers were performed using video Frenzel glasses (*VideoStar DIFRA)*. The positional tests were performed as described by Cole and Honaker (2022). Spontaneous nystagmus assessment was performed on all participants before positional tests to evaluate acute and/or central pathologies. A fast phase velocity > 4°/s was accepted as an indicator of spontaneous nystagmus in this study (Jacobson et al. 2016). During the positional tests, the presence of characteristic nystagmus, as indicated by Cole and Honaker (2022), was assessed. Based on the characteristic nystagmus observed during the maneuver, a diagnosis of benign paroxysmal positional vertigo (BPPV) was made. Additionally, even if no characteristic nystagmus was observed during the positional tests, participants were asked whether they felt subjective vertigo. Patients who described subjective vertigo during the maneuver were noted.

### 2.9 Statistical Analysis

The statistical analysis was conducted using the IBM SPSS 21 (SPSS Chicago, IL, USA) software. The normal distribution of data was assessed using the Shapiro–Wilk test. The Mann–Whitney U test was utilized to compare matched groups for normally distributed data; in other cases, the *t*‐test was employed. Categorical data were evaluated using the Fisher Exact test. The relationships between the numerical data were investigated using Spearman's correlation coefficient. A significance threshold of *p* < 0.05 was accepted for all statistical analyses.

## 3 Results

In this study, none of the children had spontaneous nystagmus. Only one child (4%) was diagnosed with posterior canal BPPV, while eight children with dizziness (31%) described subjective vertigo without positional nystagmus during the positional tests (a total of 35%). The remaining children (65%) did not exhibit positional nystagmus or subjective vertigo in the positional tests.

The average duration of dizziness among children was 8.00 ± 13.15 (range 0.25–48) months. Fifteen children (58%) had good sleep quality, while 11 children (42%) had poor sleep quality. The mean SDSC score of children was 42.69 ± 11.59. A significant positive correlation was found between the dizziness durations and SDSC scores (*p* = 0.03, *r_s_*: 0.42). In terms of subscales of the SDSC, there was a significant positive correlation only between the sleep initiation and maintenance disorders (*p* = 0.04, *r_s_* = 0.40) subscale, the excessive somnolence disorders subscale (*p* = 0.03, *r_s_* = 0.40), and dizziness duration, as shown in Table 2. The graphical abstract   summarizes the main findings of the current study. As shown in the figure of the graphical abstract, an interesting finding of the current study was that eight children (73%) with poor sleep quality had positional vertigo such as Benign Paroxysmal Vertigo of Childhood (BPVC). One of them was diagnosed with posterior canal BPPV, while the other seven reported subjective positional vertigo. Only one child (7%) with good sleep quality described subjective positional vertigo during positional testing.

**TABLE 2:** The relationship between the dizziness duration and the subscales of the Sleep Disturbance Scale for children.

Sleep disturbance scale for children	Dizziness duration		
	Median (min–max)	*r_s_*	*p*
Disorders of initiating and maintaining sleep	13 (8–24)	0.40	0.04*
Sleep breathing disorders	4 (3–9)	0.10	0.60
Disorders of arousal nightmares	4 (3–8)	0.28	0.15
Sleep wake transition disorders	8 (6–19)	0.29	0.13
Disorders of excessive somnolence	7 (5–17)	0.40	0.03*
Sleep hyperhydrosis	2 (2–7)	0.11	0.57

*Note*: *p < 0.05**;** *r_s_*: Spearman's correlation coefficient.

**FIGURE 1:**
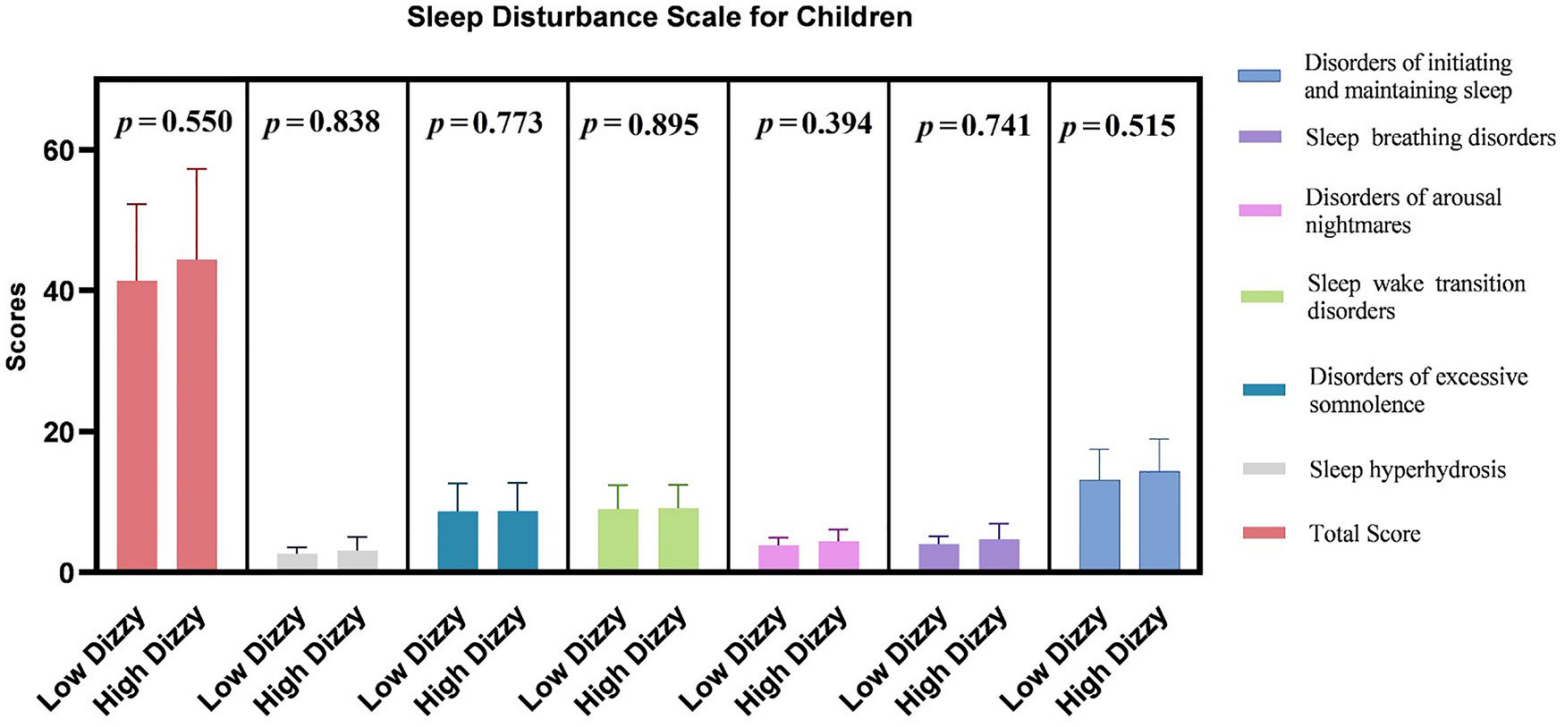
The mean total and subscale scores of the Sleep Disturbance Scale for Children were compared between children with low and high vertigo severity. The bars indicate the mean scores ± standard deviation.

The mean of VAS vertigo severity scores was 5.19±1.65. Eleven children (42%) had high vertigo severity, while 15 children (58%) had low vertigo severity. There were no significant differences between the VAS vertigo severity groups in terms of the total SDSC scores and SDSC subscales (*p* > 0.05, Figure 1).

**FIGURE 2:**
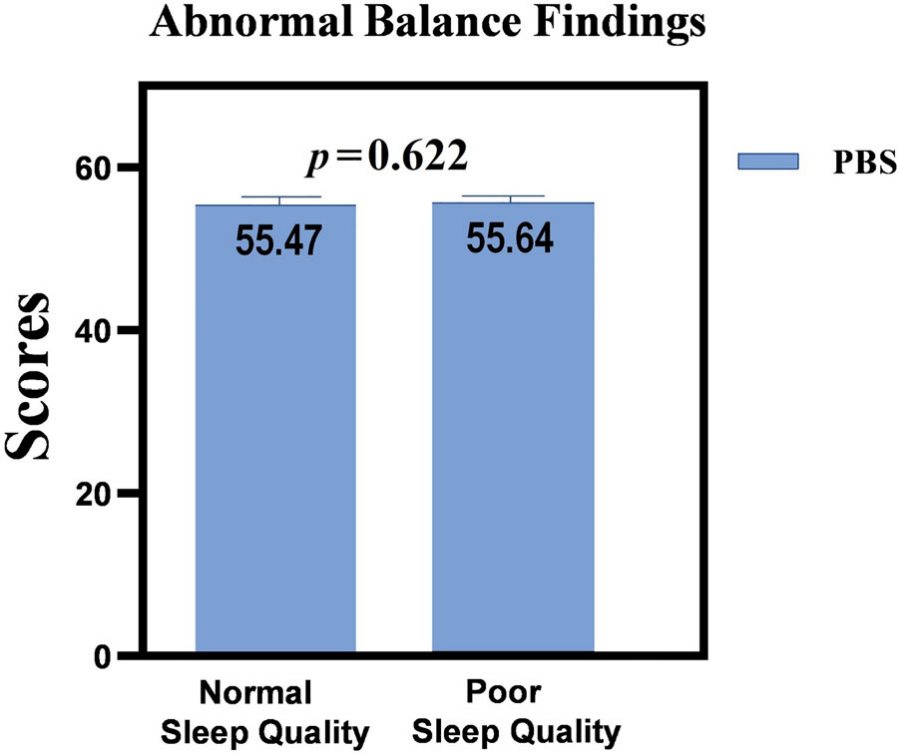
Pediatric Balance Scale scores by sleep quality groups. Mean scores on the Pediatric Balance Scale are compared between children with normal and poor sleep quality. The bars represent the mean scores ± standard deviation.

The average PBS scores were 55.54 ± 0.90. In this study, functional balance scores were not converted into categorical data because the participants' PBS scores were similar (median: 56; min–max: 53–56). Moreover, the PBS scale scores did not show a normal distribution (Shapiro–Wilk *p* < 0.001). Therefore, regarding PBS scores, there was no significant difference between the sleep quality groups (*p* > 0.05, Figure 2). In addition, there was no relationship between VAS vertigo severity (*p* = 0.44), PBS scores (*p* = 0.39), and SDSC scores.

## 4 Discussion

This study examined the relationship between sleep quality, vertigo severity, and the duration of dizziness in children experiencing dizziness. Growth and development occur during sleep. Especially in childhood, long‐lasting vestibular symptoms such as vertigo and dizziness can negatively affect the growth and development of these children by affecting their sleep quality. Segura‐Jimenez et al. (2015) conducted a study with 380 children and 304 children to investigate the relationship between daily sleep duration and health complaints, such as headache, abdominal pain, backache, toothache, falling, feeling irritable or angry, tenseness, and dizziness. They found that children who had better sleep quality reported better overall health. Additionally, they reported that children and adolescents who did not have trouble falling asleep were more likely to report no health problems (Segura‐Jimenez et al. 2015). Duarte et al. (2012) reported that individuals with vestibular symptoms and dizziness had a higher risk for sleep disorders. Furthermore, vestibular symptoms such as dizziness and vertigo can significantly impact the daily life and quality of life of both children and parents (Fancello et al. 2021). As a primary outcome of the current study, children with poor sleep quality have a longer duration of dizziness than those who sleep well. In other words, decreased sleep quality may be associated with an increase in the duration of dizziness. Moreover, it was observed that children with poor sleep quality are more predisposed to exhibit positional vertigo during the diagnostic maneuvers.

Sleep disturbances can impact cognitive and emotional functioning, but limited knowledge regarding their relationship to vestibular functioning exists. Experimental sleep deprivation is known to cause vestibular issues, such as postural instability and disruptions in visuospatial perception. Sleep deprivation in humans can affect the function of the posterior parietal cortex, which is crucial for processing vestibular input and controlling the vestibulo‐ocular reflex (VOR) (Ahrberg et al. 2012; Batuk et al. 2020; Robillard et al. 2011). In our study, we did not evaluate patients' VOR gains. Future studies could benefit from including this evaluation for a more comprehensive understanding.

On the other hand, sleep disorders can negatively affect the psychological and mental health of individuals. In the literature, some studies (Quarck et al. 2006; Furtado et al. 2016) have shown that in people with sleep problems and poor sleep quality, decreased concentration causes an increase in the need for alertness, reducing postural stability. In another study, the authors found that treating patients with chronic dizziness also helped improve the treatment of sleep disorders (Sugaya et al. 2017). Therefore, it is important to remember that treating dizziness complaints in children may impact sleep quality and development. Psychological factors were not controlled in this study, which is one of its limitations. It should be noted that psychological factors may be a confounding variable in future studies.

Structures within the inner ear are responsible for hearing and balance functions. Due to the anatomical proximity of the hearing and vestibular systems within the inner ear, a relationship between vestibular system disorders and hearing loss is known (Santos et al. 2015). Hearing loss is a risk factor for balance impairment, and these conditions often co‐occur (Melo et al. 2021). Hearing loss also causes sleep disorders (Asplund 2003; Hallam 1996). For these reasons, patients with hearing loss were not included in our study to examine the effect of balance on sleep quality. All patients in our study had normal hearing.

Peripheral vestibular problems and dizziness are more common in women (Hulse et al. 2019; Filippopulos et al. 2017). The primary cause of this is unknown; however, it is thought to be a combination of variables. Hormonal effects alone cannot explain this phenomenon (Hulse et al. 2019). Consistent with previous research, 19 (73.1%) of the children in our sample who reported experiencing vertigo or dizziness were female, and seven (26.9%) were male. Filippopulos et al. (2017) attempted to identify potential risk factors for vertigo and dizziness in adolescents aged 12–19. In our study, the age range of the children was 9–16. Some of our participants were in pre‐puberty, and others were in puberty. In line with the literature, the authors thought that the prevalence of vertigo and dizziness may increase during puberty, due to the current study (Filippopulos et al. 2017).

Neurons associated with sleep stages are located in the pontine reticular formation and the raphe nuclei, which also receive information from the otolithic organs. The connection between the vestibular nucleus and the suprachiasmatic nucleus—the primary structure responsible for regulating circadian rhythms—may contribute to this relationship (Andrade Junior et al. 2021; Drummond and Brown 2001). However, the specific sleep features related to vestibular problems are not well understood (Altena et al. 2023).

Sleep disorders are common among children and adolescents (Åslund et al. 2018). In school‐age children aged 6–11, the prevalence of sleep disorder symptoms ranges from 10% in the general population to as high as 75% in those with neurodevelopmental disorders (Quach et al. 2011; Souders et al. 2009). Research shows that the period from late childhood to early adolescence is particularly sensitive, with children in this age group at an increased risk of developing sleep problems and anxiety (McMakin and Alfano 2015). While a small percentage of children experience intrinsic sleep disorders that require medical attention, such as sleep apnea, the majority face behavioral sleep problems. These issues often present as difficulties initiating sleep, maintaining sleep, or experiencing early morning awakenings (Allen et al. 2016).

In this study, a statistically significant moderate correlation was found between the subscales of sleep initiation and maintenance disorders, excessive sleepiness disorders, and the duration of dizziness. As the duration of dizziness increased, participants experienced greater difficulty in initiating and maintaining sleep. This moderate correlation suggests that these factors may influence each other and highlights the importance of addressing both conditions in clinical practice. Sleep initiation and maintenance problems are often associated with excessive daytime sleepiness, as individuals who cannot maintain sleep at night tend to experience increased drowsiness during the day. In our study, the average duration of dizziness among participants was 8.00 ± 13.15 months (range: 0.25–48 months). While a brief period of dizziness (e.g., 1 week or 0.25 months) appeared to have a limited effect on sleep quality, prolonged dizziness was associated with a greater negative impact. This finding indicates that early intervention in children experiencing dizziness may help prevent deterioration in sleep quality. The effect of dizziness duration on sleep may be explained by the increase in psychological distress over time, which in turn can impair sleep quality. Interestingly, no significant relationship was found between dizziness duration and other SDSC subscales, suggesting that further research is needed to explore these associations. Additionally, a significant limitation of our study is the absence of a control group, which could have enhanced the strength of our findings and allowed for more precise comparisons.

On the other hand, there are limited studies on the pediatric population, although BPPV is the most common peripheral vestibular disorder in the adult population (Brodsky et al. 2018; Wiener‐Vacher et al. 2018). Wiener‐Vacher et al. (2018) reported that the prevalence of BPPV in children is less (< 1%). However, several recent studies (Brodsky et al. 2018; Balzanelli et al. 2021) have reported that the incidence of BPPV in the pediatric population is not as rare as thought. According to an extensive cohort study (110 pediatric patients aged 5–19 years) by Brodsky et al. (2018), 19.8% of pediatric patients were diagnosed with BPPV (mostly the posterior canal was affected). The gender ratio (F:M) was reported as 3:2. In addition, Brodsky et al. (2018) reported that the prevalence of BPPV by age increased during adolescence in their cohort. In contrast, Lee et al. (2017) showed that BPVC is the most common vestibular disorder in primary school children (30.1%), while vestibular migraine is the most common vestibular disorder in adolescents (30.4%). Consistent with the previous studies that reported that BPPV is a rare vestibular disorder in pediatric patients (Lee et al. 2017; Wiener‐Vacher et al. 2018; Galluzzi and Garavello 2022), only 4% of children with dizziness in this study were diagnosed with BPPV. In comparison, 31% were diagnosed with BPVC. In the literature, recurrence of BPPV was shown to be five times as common in patients with vestibular migraine or BPVC (Brodsky et al. 2018). Although the pathophysiology of BPVC is still uncertain, it was thought that BPVC may be a precursor of migraine in children (Lee et al. 2017). Another limitation of this study is that the participants were not asked whether they had a history of migraine.

The vestibular system plays a crucial role in maintaining balance and providing essential information about the position of the head in space. It functions reflexively, ensuring postural stability while standing and helping regulate sleep patterns when lying down and during sleep (Wiener and Taube 2005). By exploring the connection between head position and the initiation and duration of sleep, we can develop strategies to enhance sleep practices, ultimately contributing to better overall health and well‐being.

Generally, BPPV patients experience vertigo attacks when they change their head position in bed, which can affect their sleep quality (Iranfar and Azad 2022). Poor sleep quality has also been found to cause BPPV recurrence (Ertugrul and Soylemez 2019; Su et al. 2016). In our study, it was not questioned in detail which sleeping position and at what time of day the children's dizziness was triggered. This situation prevented us from explaining the relationship between the head positions in which the children's dizziness was triggered during sleep and their sleep quality.

Our study presents several important limitations due to its cross‐sectional design, including a small sample size, the absence of a control group, and reliance on a single assessment tool for evaluating sleep. Additionally, the reported duration of dizziness showed substantial variability (mean ± SD: 8.00 ± 13.15 months), ranging from 1 week to 4 years. This variation may be attributed to the fact that some participants experienced acute episodes of dizziness following recent vestibular insults, while others had long‐standing complaints possibly related to chronic vestibular disorders or psychosomatic factors. We acknowledge that such variability could introduce potential confounding effects. In this context, our study may be considered a pilot investigation. Future research should involve longitudinal, case‐control designs with more precisely defined sample groups (e.g., individuals with chronic dizziness) and incorporate objective measures of sleep quality for a more comprehensive understanding of the relationship between dizziness and sleep disturbances.

## 5 Conclusion

Improving sleep quality in children experiencing dizziness is crucial for alleviating symptoms of vertigo and dizziness. Future research should incorporate a detailed medical history, comprehensive vestibular testing, and sleep assessments, employing larger sample sizes. This method will significantly enhance our understanding of the condition's underlying causes and improve diagnosis and treatment.

## Author Contributions

**Busra Umame Sahin**: methodology, writing—original draft, writing—review and editing. **Tugce Gurel Soylemez**: methodology, writing—original draft, writing—review and editing, formal analysis, data collection. **Gorkem Ertugrul**: supervision, resources, methodology, writing—review and editing.

## Ethics Statement

An approval was received from local ethics committee Hacettepe University (GO 23/439).

## Consent

Written approval was received from all subjects involved in the study.

## Conflicts of Interest

The authors declare no conflicts of interest.

## Peer Review

The peer review history for this article is available at https://publons.com/publon/10.1002/brb3.70654

## Data Availability

Research data are not shared.
